# Low-dose prophylaxis protocol for heterotopic ossification after hip preservation surgery in a sport participants cohort

**DOI:** 10.1051/sicotj/2023024

**Published:** 2023-09-05

**Authors:** Matteo Olivero, Bruno Capurro, Pedro Reis-Campos, Alessandro Aprato, Olufemi Ayeni, Anuj Chawla, Ricardo Larrainzar Garijo, Oliver Marín-Peña

**Affiliations:** 1 Centro Traumatológico Ortopédico, (CTO), University of Torino 10126 Torino Italy; 2 Department of Orthopaedic Surgery and Sport Traumatology, IMSKE Hospital 46024 Valencia Spain; 3 Orthopedic and Traumatology Department, Hospital Vila Franca de Xira 2600-009 Portugal; 4 Division of Orthopaedic Surgery, Department of Surgery, McMaster University Medical Center L8S 4S4 Hamilton Canada; 5 Department of Orthopaedic Surgery, Tan Tock Seng Hospital PC 308433 Novena Singapore; 6 Department of Orthopaedic Surgery and Traumatology, Hospital Universitario Infanta Leonor 28031 Madrid Spain; 7 European Hip Preservation Associates, EHPA-ESSKA Section Europe

**Keywords:** Heterotopic ossification, Femoroacetabular impingement, Post-operative prophylaxis, Hip arthroscopy

## Abstract

*Background*: Heterotopic ossification (HO) is a well-known complication of arthroscopic and open surgical treatment of femoroacetabular impingement (FAI). Incidence of heterotopic ossification has been reported in the literature between 0% and 44% after hip arthroscopy and between 18.2% and 25% after anterior mini-open surgery. Currently, pharmacological prophylaxis with NSAIDs and selective COX-2 inhibitors are commonly used and their effectiveness is well documented in literature. *Hypothesis*: We hypothesized that the low-dose prophylaxis protocol with selective cox-2 inhibitors decreases the risk of heterotopic ossification in open or arthroscopic hip preservation surgery in athletes. *Methods*: This study is an analysis of prospectively gathered data on 98 sport participant patients who underwent arthroscopic or anterior mini-open treatment for FAI between April 2008 and April 2018. All the patients received postoperative oral prophylaxis with 60 mg etoricoxib once daily for two weeks. Post-operative X-rays were performed at 1, 3, and 12 months after surgery and reviewed by two orthopedic surgeons blinded to the type and side of surgery. HO were graded according to the Brooker classification. Descriptive statistics was used to analyze demographic data. Bivariate analysis was performed to analyze the association of HO with each of the following variables: type of surgery, physical activity, time of evolution of symptoms, age at surgery, and sex. Finally, a regression model analysis was performed to determine the presence of confounding effects between variables. *Results*: The study cohort was composed of 54 patients in the arthroscopic treatment group and 44 patients in the anterior mini-open group. HO was identified in 6 (13.6%) patients in the mini-open group. No HO was identified in the arthroscopic group. In the bivariate analysis, “type of surgery” was the only variable that showed a statistically significant association with HO (*p* = 0.007). *Conclusion*: Results of this study suggest that anterior mini-open treatment was characterized by a higher risk of HO development compared to hip arthroscopy for femoroacetabular impingement treatment regardless of pharmacological prophylaxis. The treatment regimen of 60 mg etoricoxib daily for two weeks was an effective prophylaxis for HO formation in sport participant patients compared with data available in the literature.

## Introduction

Femoroacetabular impingement (FAI), defined as a mechanical conflict between the femoral head-neck junction and the acetabular rim, is an increasingly recognized condition mostly in young and active patients [1, 2]. Possible surgical strategies include: surgical dislocation of the hip, arthroscopic treatment, and anterior mini-open technique with or without arthroscopic assistance [1, 3]. Every surgical method used to treat FAI has both advantages and disadvantages. For example, surgical hip dislocation proposed by R. Ganz [4], can be complicated by muscle injury, greater trochanter non-union, nerve-related complications, and secondary surgery for hardware removal [5]. Injury to muscles and lateral cutaneous nerves are possible complications when minimally invasive anterior approaches are used. Arthroscopic approaches can be complicated by pudendal nerve compression, fluid extravasation and, finally, is characterized by a lengthy learning curve [6, 7].

Heterotopic ossification (HO) is a well-documented complication of all of these treatments and is represented by the abnormal formation of normal bone in the soft tissues [8, 9]. Acquired HO is normally the result of trauma or major neurologic injury but the exact biological and molecular pathway of formation is still unclear [10, 11]. Some proposed theories for HO development include the transformation of mesenchymal cells activated by bone morphogenetic proteins released into soft tissues after trauma. Subsequently, inflammation promotes the differentiation of mesenchymal cells into osteoblasts and the consequent formation of ectopic bone [12, 13]. Several risk factors for HO are known including male sex, obesity, old age, previous history of HO, head and nervous system injury, lateral surgical approach to the hip, osteoarthrosis, and ankylosing spondylitis [8, 14].

The incidence of HO after hip arthroscopy has been reported in the literature between 0% and 44% [9] but most studies report incidences between 1% and 12% [12]. Fewer data is available about HO after open surgery with an anterior mini-open approach. For these patients, incidence rates range between 18.2% and 25% which are reported in the literature [6, 15].

In most cases, HO is identified radiographically and is completely asymptomatic. However, larger amounts of HO can cause impingement, decrease the range of motion, joint stiffness, pain and can lead to the necessity of revision surgeries [9, 16].

Currently, anti-inflammatory drugs (NSAIDs) and low-dose radiotherapy represent the two most widely accepted prophylaxis for HO. No clinically significant differences in the effectiveness of the two modalities have been reported in the literature. However, NSAIDs are more commonly used for lower cost and ease of administration [16, 17]. Selective cyclooxygenase 2 (COX-2) inhibitors are associated with a lower risk of gastrointestinal side effects compared to non-selective NSAIDS while maintaining the same efficacy in preventing the formation of HO after hip surgery [9, 18, 19].

This study hypothesizes that the low-dose prophylaxis protocol with selective COX-2 inhibitors decreases the risk of heterotopic ossification in open or arthroscopic hip preservation surgery in athletes.

## Methods

**Figure 1:**
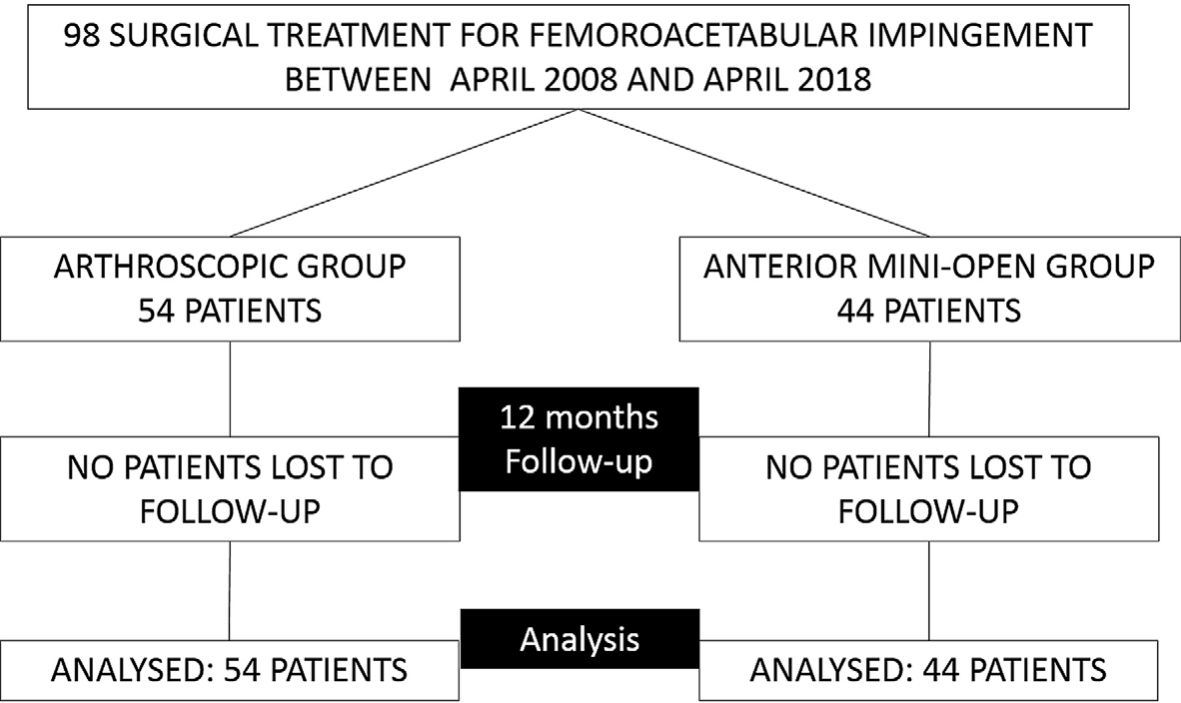
Flow diagram of patients included in the study.

The study represents a retrospective analysis of prospectively collected data. We evaluated a cohort of 98 patients who underwent treatment for FAI in a teaching orthopaedic hospital from April 2008 to April 2018 (Figure 1). The study’s protocol was approved by the local institution’s review board.

All the patients who underwent preservation hip surgery for FAI during the time of the study were included in our study. Patients were divided according to the type of surgery into an arthroscopic treatment group and an anterior mini-open treatment group. A single senior orthopaedic surgeon performed all the surgeries.

In all patients, a resection of the FAI-related bony morphology was completed. The stability of the labrum was evaluated and if deemed unstable it was repaired using suture anchors. In the arthroscopic group, Interportal capsulotomy and/or T capsulotomy were performed and closed at the completion of the case. In the mini-anterior group, a standard mini-anterior (Hueter) approach was completed followed by an arthroscopic evaluation for intra-articular damage. Labral and capsule management was the same as the arthroscopic group. Patients were informed about both types of treatment and asked about any preferences before surgery. The patient´s option was respected but when no preference was indicated, senior surgeon selected the approach with random criteria.

All the patients received standardized post-operative prophylaxis for HO. The prophylactic protocol included oral 60 mg Etoricoxib once daily for two weeks beginning the day after surgery.

The same hip postoperative rehabilitation protocol for both groups was applied. Partial weight bearing with two crutches was allowed for 3 weeks. Motion restriction above 90° of flexion and 45° abduction were recommended during the first 2 weeks. Static bike practice was included after the first week with the previously mentioned motion restrictions. Regarding sports activities, patients resumed non-impact sports at 3 months and impact sports at 6 months.

Patient were excluded from the study following these criteria: lack of information from surgical reports, evidence of previous heterotopic ossification in X-rays, previous surgical interventions on the same hip, concurrent head injury or nervous system injury, previous pelvic fracture, allergy to Etoricoxib, or follow up less than 12 months.

We collected the patient’s characteristics including sex, diagnosis, age at the time of surgery, physical activity level prior to surgery, and time to symptom development. Post-operative X-rays (anteroposterior and axial view) were performed at 6 weeks, 3 months, and 1 year from the surgery. Two orthopaedic surgeons, independently, reviewed all the preoperative and post-operative X-rays. They were blinded to the type of surgery, side of operation, and clinical outcomes of patients. Any finding consistent with HO was graded according to the Brooker et al. classification [20]: grade 0 (no ossification); grade I (islands of bone in the soft tissues around the hip); grade II (bone spurs from the pelvis or from the proximal femur with at least 1 cm between opposing bone surfaces); grade III (bone spurs from the pelvis or from the proximal femur with less than 1 cm between opposing bone surfaces) and grade IV (apparent bone ankyloses of the hip).

### Statistical analysis

Data were collected with Microsoft Excel® and subsequently analyzed with IBM SPSS® software. Descriptive statistics were used for all demographic data and the assessment of the incidence of heterotopic ossification.

Bivariate analysis was performed to analyze the association of heterotopic calcification with each of the following variables: type of surgery, physical activity level prior to surgery, time to symptom development, age at surgery, and sex. Kendall’s nonparametric correlation coefficient *τ*_b _was used to analyze two at least ordinal variables; Kruskal–Wallis (for multi-nominal variables) and Mann–Whitney (for dichotomous variables) nonparametric tests were used to analyse the correlation of an at least ordinal variable with a nominal variable. To analyze the correlation of two nominal variables, Fisher’s exact test was performed. Finally, a regression model was performed to avoid the confounding effect between variables.

## Results

In total, 98 sport participant patients were included in this study and none were lost to follow-up at 12 months. The arthroscopic FAI treatment group was composed of 54 patients (55.1%) and 44 patients (44.9%) who underwent mini-anterior FAI treatment.

The baseline data of the two groups are shown in Table 1. No significant differences were found between the two groups’ demographic data.

**Table 1:** Baseline data of study cohort.

		Arthroscopic treatment	Anterior mini-open	Total
Patients (*n*)		54 (55.1%)	44 (44.9%)	98 (100%)
Sex	Male	30 (30.6%)	32 (32.7%)	62 (63.3%)
	Female	24 (24.5%)	12 (12.2%)	36 (36.7%)
Age (years)	Median	35	39.9	37.2
	IQR	18.3–50.1	26.1–60.6	18.3–60.6
Physical activity	No sport	0 (0%)	1 (1.0%)	1 (1.0%)
	Some sport	15 (15.3%)	10 (10.2%)	25 (25.5%)
	Sport more than twice a week	39 (39.8%)	33 (33.7%)	72 (73.5%)
Time of evolution of symptoms	Since a year or less	14 (14.3%)	11 (11.2%)	25 (25.5%)
	More than a year, less than two	11 (11.2%)	5 (5.1%)	16 (16.3%)
	Since more than two years	29 (29.6%)	28 (28.6%)	57 (58.2%)

**Figure 2:**
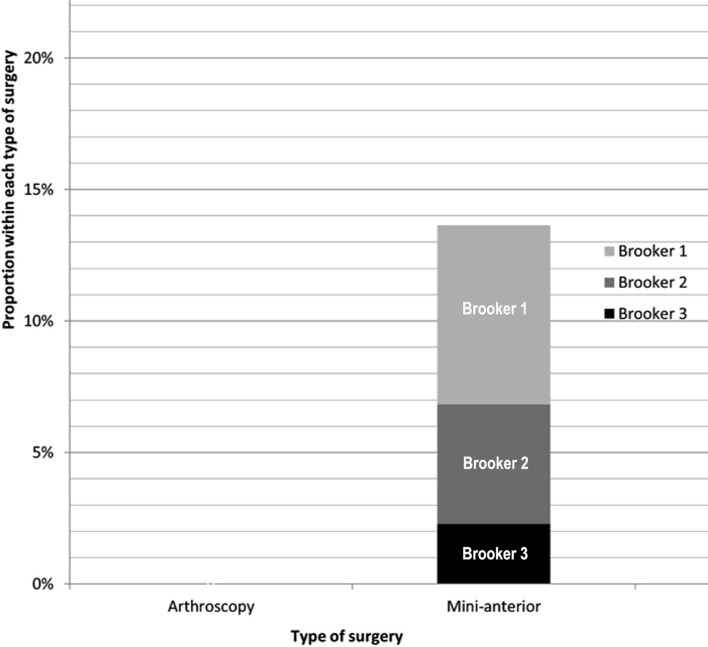
Frequencies of the different degrees of heterotopic ossification for type of surgery. Note that the heights of the bars, as indicated by the vertical axis, represent the *relative* frequencies for each type of surgery, which are not proportional to the *absolute* frequencies indicated within the bars, due to the different sample sizes of each type of surgery.

No patients in the arthroscopic treatment group developed heterotopic ossification at 12 months of radiographic follow-up. Radiographic findings of HO were detected in 6 of 44 (13.6%) patients treated with a mini-anterior approach. None of these patients were symptomatic and none required surgical intervention to remove the ossification. According to the Brooker classification, HO in three patients was classified as grade I (50%), two cases as grade II (33.3%) and one as grade III (16.7%). The distribution of HO in the population is represented in the plot in Figure 2.

In the bivariate analysis, type of surgery was the only variable that showed a significant association with the development of HO. Compared to the open treatment with a mini-anterior approach, arthroscopy shows a significantly lower Brooker score in the Mann–Whitney test with *p* = 0.005 and significantly fewer cases with calcification in Fisher’s test, with an odds ratio of 0 (95% confidence interval: 0.000 – 0.644) and *p* = 0.007. No other variables considered showed associations with the HO.

The type of surgery is the only variable that showed a significant association in the bivariate analysis. The regression analysis yielded no other or additional findings.

## Discussion

The purpose of this retrospective analysis was to evaluate the incidence of HO diagnosed with FAI treated arthroscopically or with the anterior mini-open approach in a cohort of sport participant patients. It was hypothesized that anterior mini-open treatment was characterized by a higher incidence of HO regardless of the administration of pharmacological prophylaxis. The results of this study confirmed our hypothesis as a statistically significant higher incidence of HO in the mini-open treatment was noted. Indeed, no cases of HO were identified in the arthroscopy group whereas radiographic evidence of HO was present in 6 of 44 (13.6%) patients in the anterior mini-open group. When HO was identified in mini-anterior group, 50% were classified as grade I, 33.3% as grade II, and 16.7% as grade III according to the Brooker classification [20]. All the patients with HO were asymptomatic and none needed treatment. No other variables considered in the study showed significant association with the heterotopic ossifications.

Prophylaxis aimed to inhibit HO formation after hip arthroscopy is largely addressed by pharmacological measures (NSAIDs and COX-I) and low-dose radiotherapy [12, 16, 21]. Less information is reported about prophylaxis in the anterior mini-open treatment for FAI [1, 2, 6]. Recently, low-dose radiotherapy has been reported as the most efficacious prophylaxis to prevent HO formation. However, it has a higher cost, may require additional visits and is reserved for patients with a higher risk profile [22]. The efficacy of the prophylactic treatment with NSAIDs has been proven in the literature. Beckmann et al. [23] reported that 500 mg of Naproxen twice daily for three weeks significantly reduces, but does not eliminate, the incidence of HO after hip arthroscopy. Randelli et al. [15] reported good results with various NSAIDs prophylaxis regimes. Mortensen et al. [24] reported an equivalent rate of postoperative HO after 2 or 3 weeks of Naproxen prophylaxis. Finally, Yeung et al. [16] in a recent review, reported a 4-fold decrease in the incidence of HO with NSAID prophylaxis compared to the group of patients without NSAIDs prophylaxis. However, important side effects related to long therapy with NSAIDs are known including gastrointestinal irritation, stomach ulceration, bleeding and acute kidney failure [12, 23]. To reduce gastrointestinal side effects a selective COX-2 inhibitor can be used with comparable prophylactic efficacy to traditional NSAIDs [9, 12, 14].

To the best of our knowledge a low-dose Etoricoxib prophylaxis (60 mg daily for two weeks) following FAI surgery has not been reported in the literature before. Randelli et al. [15] reported good results with the prophylactic use of Etoricoxib in arthroscopic cases, but they used a higher dose (90 mg daily for three weeks) and it was used in a very small subset of patients (15/285 patients in the treatment group).

Our results demonstrated the efficacy of the prophylaxis with low a dose of Etoricoxib both for arthroscopic and mini-open treatment with results comparable to data available in the literature. The incidence of HO in the mini-open approach is currently not well reported in all the studies and the type of approach used for the mini-open seems to be important. Chiron et al. performing an anterolateral mini-open documented that 25% of 118 cases had progressive ossification and only 10.2% were Broker type II-III [6]. When we analyze the studies that use the same technique as this anterior mini-open study, it is difficult to compare them since Bellotti et al. did not report any cases of HO without mentioning that it was one of their outcomes to be reported in the study methodology [1], and Skowronek et al. They only reported one case out of 39, which would correspond to 2.5% [2]. Therefore, it is difficult to draw a clear conclusion on the efficacy of a low dose of selective COX-2 on prophylaxis following the anterior mini-open approach. As previously described, it is important to highlight that there is a lower incidence of HO in the anterior mini-open than in the anterolateral mini-open approach and when compared by type of HO, the present study presents a lower percentage of cases with HO Broke II or III administering low dose of Etoricoxib when this complication is reported in the results of the studies.

The higher incidence of HO in the anterior mini-open group could be related to an increased soft tissue dissection in comparison to arthroscopy. Furthermore, during arthroscopic surgery, the continuous irrigation of the joint and periarticular tissue with fluid may be effective at evacuating hematoma and bone debris [8, 21]. Moreover, improvement of hip arthroscopic techniques, resulting in less muscular and capsular trauma may contribute to this decrease as well [25].

Despite the evidence for the efficacy of prophylaxis in reducing the incidence of HO, its clinical importance is still debated in the literature [16, 26, 27]. Kurz et al. [12] in their review reported that only 25% of HO post-hip arthroscopy were symptomatic. Furthermore, Dow et al. [9] did not report any significant difference in functional scores comparing patients with or without postoperative HO independently of pharmacological prophylaxis administration. Therefore, clinicians should weigh the benefits of prophylaxis, the risk of complications and the side effects of prophylaxis using medication [16]. However, patients surgically treated for FAI are usually young, active and motivated to return to sport. In this population, ossification might decrease overall satisfaction [15, 28]. A safe dose of pharmacological prophylaxis with a low-dose selective COX-2 inhibitor is a useful adjunct following surgery [19, 29].

Timing for radiographic controls was chosen based on data available in the literature reporting that 96–100% of HO were radiographically evident at 6 weeks and did not progress after 3 months postoperatively [14, 30, 31]. Consequently, our imaging protocol (AP pelvic view and hip axial view at 6 weeks, 3 months, and 1 year) could identify every HO in our cohort and is recommended.

### Study limitation

There are some limitations to our study. First, this is a retrospective study with inherent limitations regarding study methodology. However, the use of a standardized surgical approach and technique does limit variability that can impact outcomes. Next, there is no control group with no prophylaxis administration. Nonetheless, this series is comparable to others in the literature.

## Conclusion

The results of this study suggest that anterior mini-open treatment for FAI was characterized by a higher risk of HO development compared to hip arthroscopy regardless of pharmacological prophylaxis in a sport participant patients’ cohort. Pharmacological prophylaxis for HO is recommended in sport participant patients following both hip arthroscopy and mini-open treatment for FAI. Despite the development of HO, none of our patients developed symptoms from HO. Furthermore, in our study, the prophylactic regimen of oral 60 mg Etoricoxib daily for two weeks after hip surgery for FAI was an effective prophylaxis for HO formation with HO incidence comparable to data available in the literature.
